# Biventricular strain and strain rate impairment shortly after surgical repair of tetralogy of Fallot in children: A case‐control study

**DOI:** 10.1002/hsr2.613

**Published:** 2022-05-03

**Authors:** Bahar Dehghan, Alireza Ahmadi, Shima Sarfarazi Moghadam, Mohammad Reza Sabri, Mehdi Ghaderian, Chehreh Mahdavi, Mohsen Sedighi, Hamid Bigdelian

**Affiliations:** ^1^ Pediatric Cardiovascular Research Center, Cardiovascular Research Institute Isfahan University of Medical Sciences Isfahan Iran; ^2^ Trauma and Injury Research Center Iran University of Medical Sciences Tehran Iran

**Keywords:** corrective surgery, STE, TOF, ventricular dysfunction

## Abstract

**Background:**

Early biventricular dysfunction in repaired tetralogy of Fallot (TOF) children may lead to poor clinical outcomes. We aimed to assess biventricular function in TOF children before and after surgery by speckle tracking echocardiography (STE) and compare them with the controls.

**Methods:**

Twenty repaired TOF children and 20 normal children as controls were assessed by STE. Tricuspid annular plane systolic excursion (TAPSE), left ventricular ejection fraction (LVEF), biventricular strain, and strain rate were compared before and after surgery and between TOF children and controls.

**Results:**

Postoperative LVEF (*p* = 0.001), strain (*p* = 0.001), and strain rate (*p* = 0.001) for left ventricle improved significantly compared to preoperative phase. However, postoperative left ventricular strain (*p* = 0.05) and strain rate (*p* = 0.01) in TOF children were significantly impaired compared to controls. Postoperative LVEF was correlated inversely with postoperative strain rate (*r* = −0.40, *p* = 0.04). Postoperative TAPSE (*p* = 0.001), strain (*p* = 0.001), and strain rate (*p* = 0.001) for right ventricle significantly worsened when compared with the preoperative phase. Moreover, postoperative TAPSE (*p* = 0.001), strain (*p* = 0.001), and strain rate (*p* = 0.01) were significantly impaired compared to controls. Postoperative right ventricular strain rate was correlated significantly with the weight of children (*r* = 0.48, *p* = 0.02), and postoperative left ventricular strain showed significant correlations with aortic clamp time (*r* = 0.44, *p* = 0.04) and with ICU stay (*r* = −0.46, *p* = 0.04).

**Conclusion:**

Despite normal LVEF, TOF children exhibit impaired left ventricular strain and strain rate after surgery. TAPSE, strain, and strain rate for the right ventricle worsen after surgical repair. STE‐driven strain can be used to detect early ventricular dysfunction and the associated prognostic implications.

## 1 INTRODUCTION

Tetralogy of Fallot (TOF) is among the most common type of congenital cardiac anomalies typified by ventricular septal defects, overriding of the aorta, pulmonary stenosis, and hypertrophy of the right ventricle.^1^, ^2^ Although surgical correction of TOF is often required to relieve the cyanotic condition during the newborn period, it can increase the risk of morbidity and mortality, especially in high‐risk patients with small size, genetic disorders, and other comorbidities.^3^, ^4^, ^5^

Speckle tracking echocardiography (STE) technique was introduced as a scientific approach for evaluating global and regional myocardial function and has been broadly validated in various clinical settings such as evaluating ventricular function.^6^, ^7^ Advantages of STE include assessing radial and longitudinal myocardial deformation independence of the insonation angle and overcoming the requirement of manual tracking of myocardial wall segments during the cardiac circle.^8^, ^9^

In contrast with traditional echocardiography, STE provides a meticulous analysis of global and regional myocardial function.^10^ Moreover, evaluation of cardiac function by traditional echocardiography using ejection fraction has shown limitations in the diagnosis of early stages of right and left ventricular systolic dysfunction.^11^ It has been demonstrated that STE is a promising innovative approach in the early diagnosis of deteriorated right and left ventricular systolic function by measuring longitudinal strain and strain rate.^12^ Despite the potential benefits of STE, there are limited data on assessing early changes in biventricular function in pediatric with congenital cardiac anomalies, particularly surgically repaired TOF children.^13^ We conducted this study to determine early longitudinal changes in right and left ventricular function through measurement of strain and strain rate by STE in repaired TOF children.

## 2 MATERIAL AND METHODS

### 2.1 Study design and ethics

This case‐control study was carried out on TOF children admitted to our center and underwent surgical repair. The protocol of this study has been approved by the Medical Research Ethics Committee (IR.MUI.MED.REC.1397.260) at Isfahan University of Medical Science (Isfahan, Iran) and informed consent was obtained from the parents participating in the study.

### 2.2 Study population

From May 2019 to June 2020, a total of 38 TOF children underwent corrective surgery and were registered in this investigation. Inclusion criteria for the study were TOF children who underwent complete repair and had no primary palliation including Blalock–Taussig shunt or right ventricular outflow tract stenting. Also, TOF children with double outlet right ventricle, atrioventricular septal defect or Secundum atrial septal defect, genetic syndromes, and postoperative complications were excluded from the study. The sample size was calculated based on the prior study by Mohammad Nijres et al.^14^ reporting means of global circumferential left ventricular strain in repaired TOF patients (−17.24) and control group (−22.74). Hence, the estimated sample size for each group was 18 based on a type I error of 5%, power of 90%, and a formula for comparing means between two groups. Of 38 repaired TOF patients, 20 children met our criteria to enter the study, and their information including demographic, surgical, and echocardiographic data was collected, screened, and analyzed. Eighteen repaired TOF children were excluded from the study because of having primary Blalock–Taussig shunt (*n* = 8), right ventricular outflow tract stenting (*n* = 7), and other cardiac comorbidities (*n* = 2), and Down syndrome (*n* = 1). Twenty age‐matched children with innocent heart murmurs who were referred to the pediatric cardiology clinic for consultation were selected as a control group.

### 2.3 Surgical approaches

All surgical approaches were done under general anesthesia and the establishment of cardiopulmonary bypass. As we described previously, following the relief of right ventricular outflow tract obstruction by a transannular patch, the anterior portion of the ventricular septal defect was closed using a polytetrafluoroethylene patch through a short atriotomy and minimal retraction of the tricuspid valve. Afterward, the inferior part of the ventricular septal defect was closed via a limited ventriculotomy and minimal manipulation of the right ventricle.^3^

### 2.4 Echocardiography

A pediatric cardiology fellow under the supervision of an attending pediatric cardiologist performed STE for all TOF children included in the study to reduce interobserver bias. STE was performed a day before surgical repair and then 8 days after surgery. Echocardiography data were analyzed on the machine using strain software and compared with the results of controls. Optimal images were obtained through a commercially available EKO 7 diagnostic ultrasound system (Samsung Medison) equipped with 2–7 MHz probes and the frame rate was 88 frames per second. M‐mode echocardiography was done to quantify right and left ventricular functions via measuring left ventricular ejection fraction (LVEF) and tricuspid annular plane systolic excursion (TAPSE) from the apical long‐axis four‐chamber view. Longitudinal strain and strain rate for right and left ventricles were measured from the apical four‐chamber view utilizing STE. Strain is represented by the negative value, where a higher negative magnitude value represents better myocardial deformation.^15^ Accordingly, worsening in ventricular function was defined as a decrease in the magnitude of strain over time and an increase in the magnitude of strain was considered as an improvement in ventricular function.

### 2.5 Statistical analysis

All statistical analyses were performed using the SPSS 24.0 software (SPSS). Categorical variables were represented as frequency (%) and continuous variables as mean ± standard deviation. Kolmogorov–Smirnov test was applied to control whether data had a normal distribution or not. Categorical data were analyzed by *χ* ^2^ test and Fisher's exact test where appropriate. Paired *t*‐test was applied to compare pre‐ and postoperative variables and an unpaired *t*‐test was applied to compare continuous data between two groups of study. Correlations between LVEF and TAPSE with strain and strain rate for right and left ventricles were calculated as Pearson's correlation coefficients. *p* Value was considered statistically significant when it was less than 0.05.

## 3 RESULT

### 3.1 Demographic data and patient characteristics

Twenty TOF children and 20 controls were screened and enrolled in this study. Comparisons between TOF children and controls are summarized in Table 1. TOF children consisted of 12 males and 8 females, whereas the controls consisted of 13 males and 7 females. STE data of one male TOF child was excluded from the final analysis because he experienced postoperative complete heart block and received a permanent pacemaker. There were no statistically significant differences in age, weight, body surface area, systolic blood pressure, and diastolic blood pressure between the two groups of study. Preoperative O_2_ saturation was significantly lower in TOF children than controls (89.80 ± 2.09% vs. 95.70 ± 1.95%, *p* = 0.001).

**Table 1:** Comparison of patients' variables between two groups of study

Variable^a^, ^b^	TOF (*n* = 20)	Controls (*n* = 20)	*p* value
Age (month)^a^	19.30 ± 6.81	16.30 ± 2.71	0.075
Gender (%)^b^			
Female	12 (60)	13 (65)	0.744
Male	8 (40)	7 (35)	
Weight (kg)^a^	11.45 ± 4.29	10.80 ± 0.89	0.512
BSA (m^2^)^a^	0.31 ± 0.10	0.33 ± 0.07	0.539
SBP (mmHg)^a^	90.50 ± 3.94	91.75 ± 4.43	0.352
DBP (mmHg)^a^	60.90 ± 3.14	61.70 ± 3.24	0.433

Abbreviations: BSA, body surface area; DBP, diastolic blood pressure; SBP, systolic blood pressure; TOF, tetralogy of Fallot.

^a^ Continuous data are presented as mean ± standard deviation and analyzed using *t*‐test.

^b^ Categorical data are presented as frequency (percentage) and analyzed using χ^2^ and Fisher's exact test.

### 3.2 Operative and postoperative outcomes

In the TOF children undergoing a total correction, the mean duration of cardiopulmonary bypass and aortic cross‐clamp times were 128 ± 32 and 91.20 ± 20.6 min, respectively. Before surgery, the mean oxygen saturation was 89.80 ± 2.09% which increased significantly to 95.20 ± 1.36% after surgical repairer (*p* = 0.001). Intraoperative and postoperative outcomes are summarized in Table 2.

**Table 2:** Operative and postoperative outcomes in TOF children

Cardiopulmonary bypass time (min)^a^	128 ± 32
Aortic cross‐clamp time (min)^a^	91.20 ± 20.6
Intubation time (hour)^a^	26.08 ± 2.1
Inotrope support (day)^a^	2.6 ± 0.08
ICU stay (day)^a^	5.78 ± 0.32
Hospital stay (day)^a^	14.38 ± 5.54
Residual VSD (%)^b^	8 (40%)
Residual PS (%)^b^	13 (65%)
Complete heart block (%)^b^	1 (5%)
Permanent pacemaker implantation (%)^b^	1 (5%)
Pleural effusion (%)^b^	3 (15%)

Abbreviations: ICU, intensive care unit; PS, pulmonary stenosis; TOF, tetralogy of Fallot; VSD, ventricular septal defect.

^a^ Continuous data are presented as mean ± standard deviation and analyzed using a *t*‐test.

^b^ Categorical data are presented as frequency (percentage) and analyzed using *χ* ^2^ and Fisher's exact test.

### 3.3 Left ventricular performance

**Figure 1:**
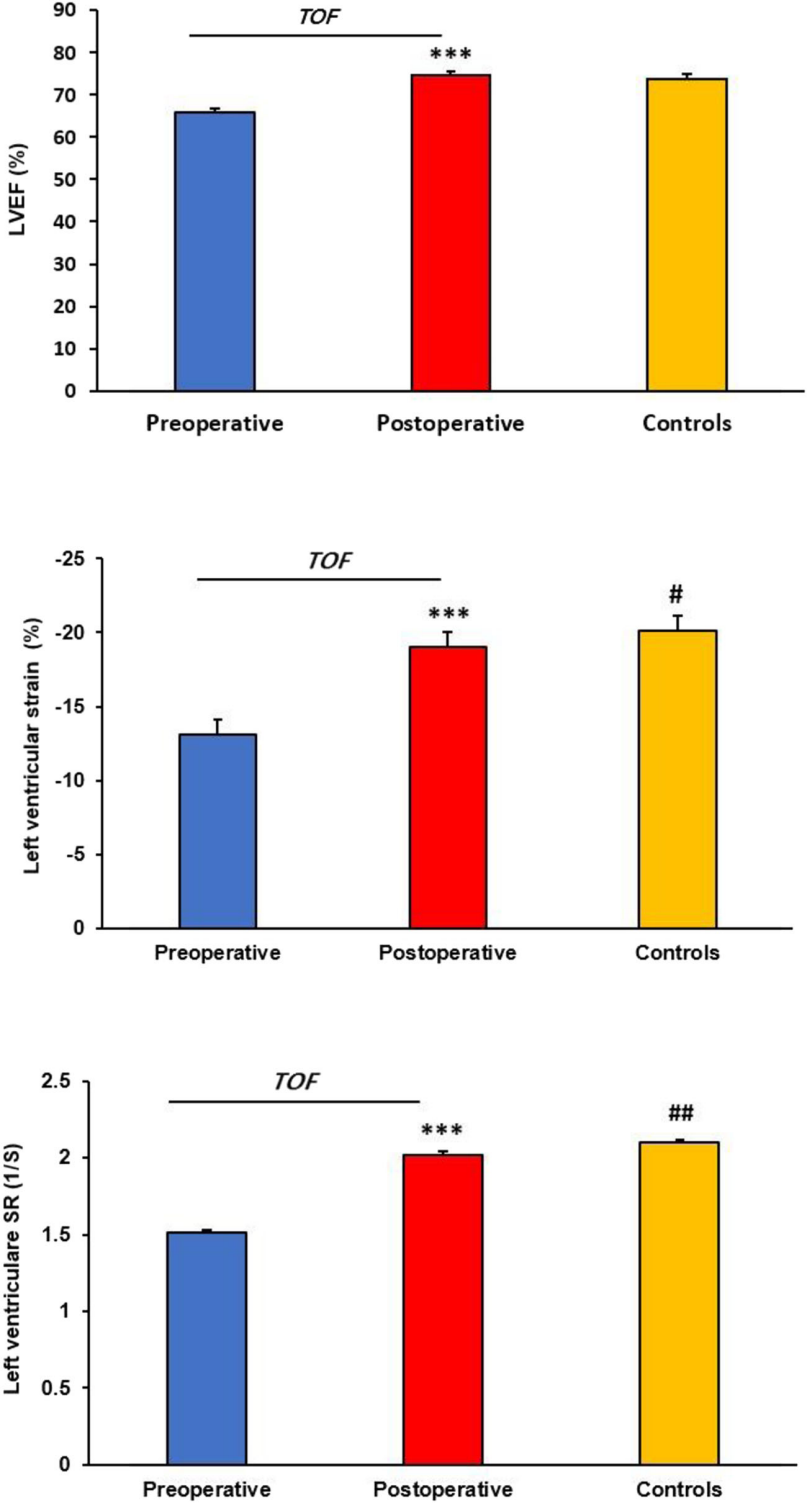
Comparison of LVEF, strain, and strain rate for left ventricle in TOF children (*n* = 19) and controls (*n* = 20) measured by speckle‐tracking echocardiography (STE). LVEF, left ventricular ejection fraction; SR, strain rate; TOF, tetralogy of Fallot. ***p < 0.001 (in comparison with preoperative); ^#^ *p* < 0.05, ^##^ *p* < 0.01 (in comparison with postoperative)

The LVEF, strain, and strain rate for the TOF children and controls are shown in Figure 1. LVEF (68 ± 4.42 vs. 74.89 ± 3.07, *p* = 0.001), strain (−13.03 ± 2.62 vs. −19.07 ± 2.18, *p* = 0.001), and strain rate (−1.51 ± 0.11 vs. −2.02 ± 0.05, *p* = 0.001) improved significantly after surgical repair in TOF children. However, postoperative strain (−19.07 ± 2.18 vs. −20.11 ± 0.95, *p* = 0.05) and strain rate (−2.02 ± 0.05 vs. −2.10 ± 0.08, *p* = 0.01) were impaired significantly in repaired TOF children compared to controls. Postoperative LVEF in TOF children was correlated inversely with postoperative strain rate (*r* = −0.40, *p* = 0.04) (Table 3). Moreover, postoperative left ventricular strain was correlated positively with aortic cross‐clamp time (*r* = 0.44, *p* = 0.04) and inversely with ICU stay (*r* = 0.46, *p* = 0.04) in TOF children (Table 4).

**Table 3:** Pearson's correlation coefficients of ventricular parameters with strain and strain rate in TOF children

Ventricular parameters (A)		Strain (B)	Correlation (A and B)		Strain rate (C)	Correlation (A and C)	
(Preoperative) LVEF	68 ± 4.42	− 13.03 ± 2.62	*r* = −0.21	*p* = 0.38	−1.51 ± 0.11	*r* = −0.45	*p* = 0.06
(Preoperative) TAPSE	1.62 ± 0.18	− 18.09 ± 1.46	*r* = −0.26	*p* = 0.27	−1.86 ± 0.13	*r* = −0.01	*p* = 0.97
(Postoperative) LVEF	74.89 ± 3.07	− 19.07 ± 2.18	*r* = −0.04	*p* = 0.84	−2.02 ± 0.05	*r* = −0.40	*p* = 0.04^a^
(Postoperative) TAPSE	1.10 ± 0.26	− 13.77 ± 1.35	*r* = 0.27	*p* = 0.25	−1.33 ± 0.19	*r* = −0.19	*p* = 0.42

Abbreviations: LVEF, left ventricular ejection fraction; TAPSE, tricuspid annular plane systolic excursion; TOF, tetralogy of Fallot.

^a^ Correlation was significant at *p* < 0.05 level.

**Table 4:** Pearson's correlation coefficients between patient characteristics and postoperative ventricular parameters in TOF children

Variable	LV strain		LV strain rate		RV strain		RV strain rate	
Age	*r* = 0.22	*p* = 0.33	*r* = 0.15	*p* = 0.51	*r* = 0.38	*p* = 0.09	*r* = 0.35	*p* = 0.12
Weight	*r* = 0.20	*p* = 0.39	*r* = −0.16	*p* = 0.49	*r* = −0.29	*p* = 0.20	*r* = 0.48	*p* = 0.02^a^
CPB time	*r* = 0.41	*p* = 0.06	*r* = −0.03	*p* = 0.87	*r* = −0.07	*p* = 0.73	*r* = 0.24	*p* = 0.29.29
ACC time	*r* = 0.44	*p* = 0.04^a^	*r* = −0.08	*p* = 0.97	*r* = −0.10	*p* = 0.67	*r* = 0.23	*p* = 0.19
Intubation time	*r* = −0.14	*p* = 0.54	*r* = 0.24	*p* = 0.29	*r* = −0.23	*p* = 0.31	*r* = −0.30	*p* = 0.19
ICU stay	*r* = −0.46	*p* = 0.04^a^	*r* = −0.25	*p* = 0.28	*r* = −0.03	*p* = 0.87	*r* = −0.30	*p* = 0.19

Abbreviations: ACC, aortic cross‐clamp; CPB, cardiopulmonary bypass; ICU, intensive care unit; LV, left ventricle; RV, right ventricle; TOF, tetralogy of Fallot.

^a^ Correlation was significant at *p* < 0.05 level.

### 3.4 Right ventricular performance

**Figure 2:**
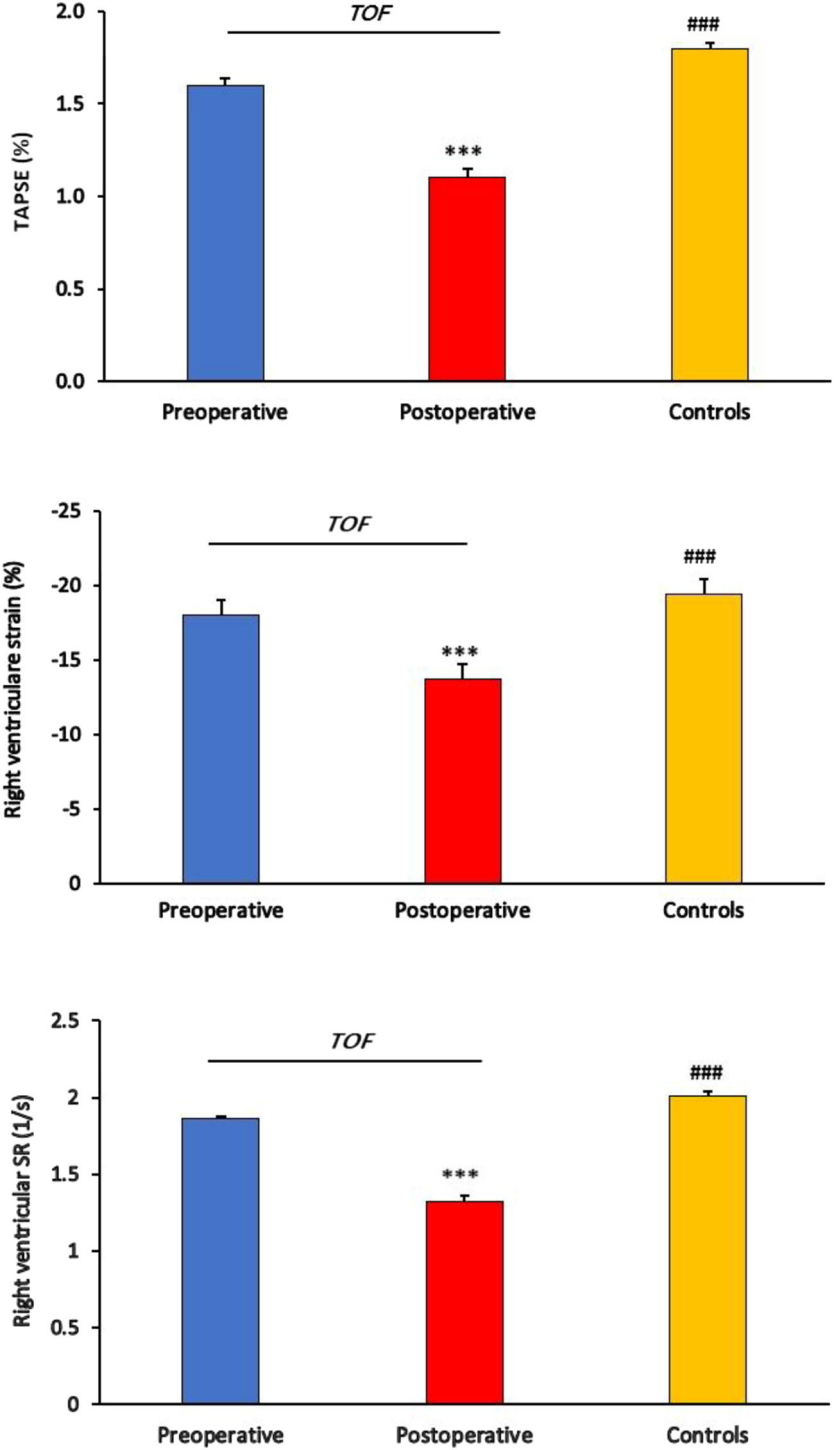
Comparison of TAPSE, strain, and strain rate values for right ventricle in TOF children (*n* = 19) and controls (*n* = 20) measured by speckle‐tracking echocardiography (STE). SR, strain rate; TAPSE, tricuspid annular plane systolic excursion; TOF, tetralogy of Fallot. ***p < 0.001 (in comparison with preoperative); ^###^ *p* < 0.001 (in comparison with postoperative)

Figure 2 presents TAPSE, strain, and strain rate measured for the right ventricle. In comparison with the preoperative phase, measured value for TAPSE (1.64 ± 0.20 vs. 1.10 ± 0.25, *p* = 0.001), strain (−17.99 ± 1.49 vs. −13.71 ± 1.35, *p* = 0.001), and strain rate (−1.86 ± 0.12 vs. −1.32 ± 0.19, *p* = 0.001) got worse after surgery in TOF children. Likewise, postoperative value for TAPSE (1.87 ± 0.16 vs. 1.10 ± 0.25, *p* = 0.001), strain (−19.38 ± 0.80 vs. –13.71 ± 1.35, *p* = 0.001), and strain rate (−2.01 ± 0.15 vs. −1.32 ± 0.19, *p* = 0.01) in TOF children was significantly impaired in comparison with controls. Our findings did not show a significant correlation between TAPSE, strain, and strain rate for the right ventricle (Table 3). However, postoperative right ventricular strain rate was correlated positively with the weight of TOF children (*r* = 0.48, *p* = 0.02) (Table 4).

## 4 DISCUSSION

This study provides preliminary results of the STE technique for the assessment of biventricular function in TOF children before and after surgical repair. Our findings indicated a remarkable improvement in left ventricular systolic function following surgical repair in TOF children. Proposed mechanisms for this change in left ventricular performance include an increase in left ventricular filling and improvement of left ventricular systolic function through ventricular interdependence.^15^, ^16^ Nevertheless, despite preserved postoperative LVEF, impaired strain and strain rate for left ventricle was found in our TOF children compared to controls, concerning early signs of ventricular dysfunction after surgery. Moreover, postoperative TAPSE, strain, and strain rate for right ventricle in TOF children were significantly impaired compared to controls. Current findings in our study showed that right and left ventricular strain and strain rates are impaired early after surgical repair in TOF children. Therefore, strain and strain rate measured by STE can be used to diagnose early ventricular dysfunction following TOF surgical repair.

Although LVEF in our TOF children improved significantly following surgery, postoperative strain and strain rate for the left ventricle were impaired compared to controls, indicating inadequate development of the left ventricle in TOF children after surgical repair. The cause of this dysfunction is a combined effect of local elements such as hypoxia and global elements such as altered left ventricular geometry due to right ventricular enlargement secondary to pulmonary regurgitation.^17^ Right ventricular myocardial fibrosis has been presented mainly in children with repaired TOF and changes in the myocardial fiber arrangement inside the right ventricular wall including oblique orientation of the longitudinal layer and the presence of a middle circumferential layer contribute to right ventricular dysfunction.^18^ Furthermore, pressure overload can induce right ventricular fiber orientation changes with a comparative dominance of the circumferential fiber direction inside the right ventricular wall.^19^ Accordingly, radial shortening, commonly produced by the circumferential fibers in the subepicardial layer of the ventricular wall, may reflect right ventricular function better than longitudinal shortening in TOF children.^20^

In addition to changes in myocardial architecture, other factors including hypertrophy and hypoxic condition before corrective surgery, probable intraoperative myocardial damage, and pulmonary regurgitation following surgical repair contribute to postoperative ventricular dysfunction. It has been shown that transventricular incision for closing ventricular septal defect and relief of right ventricular outflow tract obstruction in TOF children increases the rate of late right ventricular dysfunction and worsens the long‐term clinical outcomes.^1^, ^14^ Furthermore, severe pulmonary regurgitation can put a volume overload on the right ventricle that may adversely affect the right ventricle systolic function. However, this phenomenon does not always happen due to anatomical and functional characteristics of the right ventricle in TOF children. The type of reconstruction such as transannular patch may also affect right ventricular function because it results in the dissection of the outflow tract muscles.^21^, ^22^

TOF children in our study experienced biventricular strain and strain rate impairment early after surgery which is consistent with Nijres et al.'s results, reporting atypical segmental and global left ventricular longitudinal and circumferential strain in asymptomatic surgically repaired TOF children despite having normal LVEF.^14^ Moreover, Li et al. reported impaired right ventricular longitudinal strain and strain rate in repaired TOF children through the second postoperative year but left ventricular longitudinal strain and strain rate are close to the controls.^22^ Weidemann et al. report has shown abnormal function in regional biventricular systolic myocardial function in asymptomatic repaired TOF children that are related to irregular electrical depolarization in the right ventricle.^23^ Furthermore, a recent study in the assessment of biventricular function utilizing tissue Doppler imaging and speckle tracking has shown a significant decrease in left interventricular septum strain shortly after TOF surgery, but not in the fractional shortening.^21^ Although ventricular strain worsens early after surgical repair, DiLorenzo et al. have demonstrated that strain recovers through two postoperative years but long‐term ventricular dysfunction may begin early after surgery.^24^

Our findings revealed a significant correlation between left ventricular strain and aortic cross‐lamp time and cardiopulmonary bypass. Prior investigations demonstrated a relationship between prolonged cardiopulmonary bypass and cross‐clamp times with increased markers of diffuse myocardial fibrosis and ventricular dysfunction.^25^ These findings indicate the importance of optimal cardioprotection and minimizing the duration of cardiopulmonary bypass and aortic cross‐clamp times to reduce myocardial injury after TOF surgery. From a pathophysiological point of view, it has been shown that acute restrictive right ventricular physiology after TOF surgery is associated with severe iron loading of transferrin and postsurgical oxidative stress due to cardiopulmonary bypass.^26^ Furthermore, we found a significant correlation between postoperative ventricular strain and weight of TOF children that is consistent with Alsoufi et al. results, indicating that greater weight at the time of surgical repair is associated with strain recovery and low birth weight at surgery is linked to the adverse outcomes in infants with the ventricular disease.^27^ In addition, our result showed a correlation between postoperative left ventricular strain and ICU stay in TOF children, demonstrating that a longer length of ICU and hospital stay leads to the worsening in myocardial strain. Moreover, longer intensive care support has been shown to result in restrictive physiology after surgical repair in TOF children.^28^

Results of STE data analysis in our study have shown early biventricular dysfunction shortly after surgical repair in TOF children. Therefore, STE can be used as a useful clinical method to monitor biventricular systolic function and diagnose ventricular dysfunction in the early stages of the disease.^29^, ^30^ Although utilizing STE for longitudinal follow‐up in pediatrics, especially children with congenital cardiac anomalies, is controversial, it seems to be a promising and accurate method for evaluating the regional myocardial function and a better understanding of biventricular performance.

The present study has potential limitations. Our investigation was a single‐center study and the main limitations of our investigation were short‐term follow‐up and small sample size. Hence, we would reassess these measures within 3, 6, and 12 months following corrective surgery. Also, further studies with a larger sample size are required to validate the use of STE‐derived strain and strain rate for quantifying biventricular myocardial function in TOF children. Furthermore, our study included children undergoing total correction TOF using a transannular patch who were noncyanotic, and different surgical techniques may affect postoperative biventricular function. Therefore, our findings may not be generalizable to all TOF children repaired via the transannular patch.

Our findings indicate that surgically repaired TOF children exhibit impaired strain and strain rate for the left ventricle calculated by STE despite having normal LVEF, suggesting subclinical damage to the left ventricle systolic function. Moreover, right ventricular strain, strain rate, and TAPSE worsen early after a surgical repair, which may be a direct result of surgical insult. STE‐derived strain and strain rate can be utilized to detect subclinical biventricular dysfunction and the associated prognostic implications.

## AUTHOR CONTRIBUTIONS

**Bahar Dehghan**: Conceptualization; investigation; methodology; project administration; supervision; writing–review and editing. **Alireza Ahmadi**: Conceptualization; investigation; methodology; project administration; software; supervision; writing–review and editing. **Shima Sarfarazi Moghadam**: Conceptualization; data curation; formal analysis; investigation; methodology; project administration; software; validation; writing–review and editing. **Mohammad Reza Sabri**: Conceptualization; investigation; methodology; project administration; software; supervision; validation; writing–review and editing. **Mehdi Ghaderian**: Conceptualization; investigation; methodology; project administration; software; supervision; writing–review and editing. **Chehreh Mahdavi**: Conceptualization; data curation; investigation; methodology; project administration; writing–review and editing. **Mohsen Sedighi**: Conceptualization; data curation; formal analysis; methodology; software; writing–original draft; writing–review and editing. **Hamid Bigdelian**: Conceptualization; data curation; investigation; methodology; validation; writing–review and editing.

## CONFLICTS OF INTEREST

The authors declare no conflicts of interest.

## TRANSPARENCY STATEMENT

The lead author (manuscript guarantor) affirms that this manuscript is an honest, accurate, and transparent account of the study being reported; that no important aspects of the study have been omitted; and that any discrepancies from the study as planned (and, if relevant, registered) have been explained.

## Data Availability

Related data of this project are available on request.
